# Polyvinylpyrrolidone-Functionalized NiCo_2_O_4_ Electrodes for Advanced Asymmetric Supercapacitor Application

**DOI:** 10.3390/polym17131802

**Published:** 2025-06-28

**Authors:** Rutuja U. Amate, Mrunal K. Bhosale, Pritam J. Morankar, Aviraj M. Teli, Chan-Wook Jeon

**Affiliations:** 1 School of Chemical Engineering, Yeungnam University, 280 Daehak-ro, Gyeongsan 712-749, Republic of Korea; rutu.nanoworld@gmail.com (R.U.A.); mrunal.snst.1@gmail.com (M.K.B.); pritam.nanoworld@gmail.com (P.J.M.); 2 Division of Electronics and Electrical Engineering, Dongguk University-Seoul, Seoul 04620, Republic of Korea; avteli.teli@gmail.com

**Keywords:** NiCo_2_O_4_ nanosheets, polyvinylpyrrolidone, hydrothermal synthesis, electrochemical energy storage, asymmetric supercapacitor

## Abstract

Designing advanced electrode architectures with tailored morphology and redox synergy is essential for achieving high-performance supercapacitive energy storage. In this study, a PVP-assisted hydrothermal approach was employed to synthesize binder-free NiCo_2_O_4_ nanostructured electrodes directly on nickel foam substrates. By modulating the PVP concentration (0.5–2 wt%), hierarchical flower-like nanosheets were engineered, with the NiCo-P_1_ sample (1 wt% PVP) exhibiting an optimized structure, superior electroactive surface area, and enhanced ion accessibility. Comprehensive electrochemical analysis revealed that NiCo-P_1_ delivered an outstanding areal capacitance of 36.5 F/cm^2^ at 10 mA/cm^2^, along with excellent cycling stability over 15,000 cycles with 80.97% retention. Kinetic studies confirmed dominant diffusion-controlled redox behavior with high OH^−^ diffusion coefficients and minimal polarization. An asymmetric pouch-type supercapacitor device (NiCo-P_1_//AC) exhibited a wide operating window of 1.5 V, achieving a remarkable areal capacitance of 187 mF/cm^2^, energy density of 0.058 mWh/cm^2^, and capacitive retention of 78.78% after 5000 cycles. The superior performance is attributed to the synergistic integration of mixed-valence Ni and Co species, engineered nanosheet morphology, and low interfacial resistance. This work underscores the significance of surfactant-directed design in advancing cost-effective, high-performance electrodes for next-generation flexible energy storage technologies.

## 1. Introduction

The global push toward decarbonization and energy decentralization has intensified the demand for robust, efficient, and high-capacity energy storage systems. Electrochemical energy storage technologies have consequently emerged as critical enablers in bridging intermittent renewable energy generation with stable energy supply, supporting both mobile electronics and stationary grid-scale systems [1,2,3]. Among these technologies, electrochemical capacitors, commonly known as supercapacitors, have attracted considerable interest due to their exceptional power density, rapid charge–discharge characteristics, and superior operational safety compared to conventional batteries [1,4,5,6,7]. However, a primary limitation of supercapacitors is their relatively low energy density, which constrains their application in systems requiring sustained energy output [8,9,10]. To overcome this constraint, asymmetric supercapacitors have been proposed as a promising architecture. These devices combine a battery-type faradaic electrode with a capacitor-type electric double-layer electrode, thereby integrating distinct charge storage mechanisms to achieve both high energy and power densities [11,12,13]. The performance of such devices is largely dictated by the properties of the electrode materials, particularly their redox activity, charge transport dynamics, and long-term structural integrity [14]. Within this context, spinel-phase nickel cobaltite (NiCo_2_O_4_) has emerged as a leading pseudocapacitive material. Its crystallographic framework accommodates multiple valence states, namely Ni^2+^/Ni^3+^ and Co^2+^/Co^3+^, which facilitate multielectron redox transitions and enable enhanced faradaic charge storage [14]. In addition, NiCo_2_O_4_ exhibits superior intrinsic electrical conductivity in comparison to many monometallic oxides, a property that is attributed to the synergistic interaction between nickel and cobalt ions in the spinel lattice. Despite these advantages, the electrochemical performance of NiCo_2_O_4_ is highly dependent on its microstructural attributes, including morphology, crystallinity, and specific surface area, all of which are strongly influenced by the synthetic route employed [15].

A growing frame of literature underscores the critical role of synthesis methodology in optimizing NiCo_2_O_4_ performance. For instance, Zhu and co-workers reported a specific capacitance of 1254 F/g using a sol–gel method with oxalic acid, attributing the enhanced performance to improved porosity and active surface area [16]. Similarly, Yewale et al. utilized a hydrothermal method, producing microrod structures with high aspect ratios and achieving a remarkable capacitance of 1671 F/g [17]. Liu et al. demonstrated that hierarchical NiCo_2_O_4_@ NiCo_2_O_4_ core–shell nanoflakes yielded an areal capacitance of 2.20 F/cm^2^, further validating the importance of morphological engineering in maximizing electrochemical output [18]. In recent years, polymer-assisted synthesis has gained attention as a powerful strategy for morphological control of nanostructured transition metal oxides. Polyvinylpyrrolidone (PVP), a nonionic amphiphilic polymer, has been widely employed as a structure-directing agent due to its ability to coordinate metal ions through its pyrrolidone group. This coordination can modulate nucleation rates and crystal growth pathways, leading to the formation of nanostructures with uniform particle size, high porosity, and reduced agglomeration. Moreover, the amphiphilic nature of PVP enhances the dispersion of precursor species in solution, resulting in improved homogeneity and electrolyte–electrode interaction, both of which are critical for high-performance supercapacitors [19,20,21,22,23].

Although the utility of PVP has been demonstrated in the synthesis of various oxide-based composites and hybrid systems, its specific role in tailoring the structure and electrochemical properties of monolithic NiCo_2_O_4_ remains insufficiently explored. For example, Ulisso et al. fabricated a NC-LDH@ NiCo_2_O_4_ core–shell system with a capacitance of 2222 F/g and excellent retention characteristics [22], while Park et al. developed a PVP–polyaniline–NiCo_2_O_4_ hybrid achieving 698.44 μAh/cm^2^ and 86% retention, attributing the results to enhanced conductivity and interfacial compatibility [23]. Huang and colleagues also reported an energy density of 20.10 Wh/kg in NiCo_2_O_4_/N-rGO composites, highlighting the value of nanostructure control and interface engineering [24]. However, these studies predominantly focused on multi-component systems, leaving the mechanistic role of PVP in pure-phase NiCo_2_O_4_ relatively unaddressed. In light of this research gap, the present study focuses on the controlled hydrothermal synthesis of PVP-assisted NiCo_2_O_4_ nanostructures. By systematically varying the concentration of PVP in the precursor solution, we investigate its influence on particle morphology, surface properties, and electrochemical performance. The synthesized materials were characterized using X-ray diffraction (XRD), field-emission scanning electron microscopy (FESEM), and X-ray photoelectron spectroscopy (XPS) to elucidate structural and compositional changes. Electrochemical performance was evaluated through cyclic voltammetry (CV), galvanostatic charge–discharge (GCD), and electrochemical impedance spectroscopy (EIS). This work aims to establish a comprehensive understanding of the role of polymer-mediated synthesis in enhancing the electrochemical behavior of NiCo_2_O_4_, providing a rational framework for the development of next-generation pseudocapacitive materials.

## 2. Materials and Methods

### 2.1. Materials

Nickel nitrate hexahydrate (Ni(NO_3_)_2_·6H_2_O), cobalt nitrate hexahydrate (Co(NO_3_)_2_·6H_2_O), sodium hydroxide (NaOH), and polyvinylpyrrolidone (PVP, MW ~40,000) were purchased from Sigma-Aldrich, St. Louis, MO, USA and used without further purification. Deionized (DI) water was used for all solution preparations.

### 2.2. Synthesis of NiCo_2_O_4_ Electrodes

**Figure 1:**
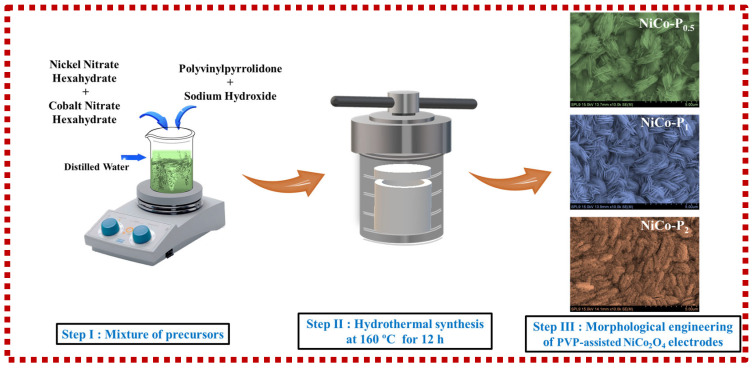
Schematic representation of the PVP-assisted hydrothermal synthesis and morphological evolution of NiCo-P electrodes.

NiCo_2_O_4_ nanostructures were synthesized on nickel foam using a hydrothermal method. Typically, 0.01 mol of Ni(NO_3_)_2_·6H_2_O and 0.02 mol of Co(NO_3_)_2_·6H_2_O were dissolved in 100 mL of DI water under constant stirring. Following complete dissolution, a designated amount of PVP (0.5 wt%, 1 wt%, or 2 wt%) was added to the solution and stirred thoroughly to ensure uniform dispersion. Subsequently, 0.06 mol of NaOH was gradually added to adjust the pH to ~11. Prior to use, nickel foam substrates were cleaned by sequential ultrasonication in 3 M HCl, ethanol, and DI water for 15 min each to remove surface oxides and contaminants. The cleaned foams were immersed in the precursor solution and placed into a 100 mL Teflon-lined stainless-steel autoclave (iNexus, Inc., Suwon, Republic of Korea). The hydrothermal reaction was carried out at 160 °C for 12 h. After naturally cooling to room temperature, the resulting samples were rinsed with DI water and ethanol, dried at 80 °C, and finally annealed at 300 °C in air for 3 h to obtain crystalline NiCo_2_O_4_ structures. The resulting electrodes were labeled as NiCo-P_0.5_, NiCo-P_1_, and NiCo-P_2_, corresponding to 0.5 wt%, 1 wt%, and 2 wt% PVP concentrations used during synthesis. A schematic illustration of the synthesis procedure for PVP-assisted NiCo_2_O_4_ electrodes is shown in Figure 1.

### 2.3. Sample Characterization and Electrochemical Measurements

XRD analysis was performed using a PAN-alytical diffractometer (Malvern Panalytical, Malvern, UK) with Cu Kα radiation to examine the crystallographic structure and phase purity of the NiCo-P electrodes. Patterns were recorded over a 2θ range of 10–90° to confirm crystallinity and phase composition. Surface morphology and elemental distribution were characterized using FE-SEM (S4800, Hitachi, Tokyo, Japan) coupled with energy-dispersive X-ray spectroscopy (EDS). Prior to imaging, electrodes were sputter-coated with platinum to minimize charging effects. FE-SEM provided microstructural details, while EDS enabled elemental mapping. XPS was conducted using a K-Alpha system (Thermo Scientific, Oxford, UK) with Al Kα radiation to determine the surface composition and oxidation states of elements in the NiCo-P electrodes. Electrochemical measurements were performed using a Biologic WBCS3000 workstation(BioLogic, Seyssinet-Pariset, France) in a three-electrode setup, with NiCo-P as the working electrode, Pt as the counter electrode, and Ag/AgCl as the reference. A 2 M KOH solution was used as the electrolyte to evaluate specific capacitance, cycling stability, and charge–discharge behavior.

## 3. Results and Discussion

### 3.1. X-Ray Diffraction Elucidation

**Figure 2:**
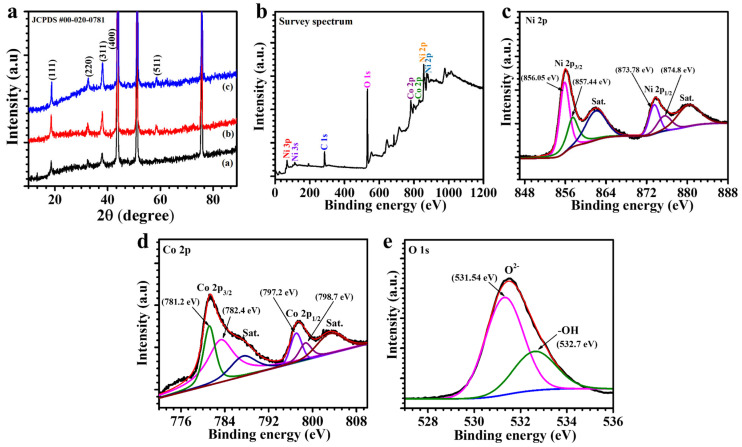
(**a**) XRD patterns confirming the cubic spinel phase of NiCo-P electrodes, (**b**) XPS survey spectrum, (**c**) Ni 2p, (**d**) Co 2p, and (**e**) O 1s high-resolution XPS spectra.

The structural properties of NiCo_2_O_4_ electrodes synthesized with varying concentrations of PVP were examined using XRD, as presented in Figure 2a. All three samples, namely NiCO-P_0.5_, NiCO-P_1_, and NiCO-P_2_, exhibit distinct diffraction peaks at 2θ values of approximately 18.9°, 31.1°, 36.7°, 44.6°, and 59.1°. These reflections are attributed to the (111), (220), (311), (400), and (511) crystallographic planes, respectively, confirming the formation of a well-defined cubic spinel NiCo_2_O_4_ phase. The observed diffraction pattern aligns well with the standard reference provided by JCPDS card number 00 020 0781 [25]. These peaks confirm the formation of a single-phase, face-centered cubic (FCC) NiCo_2_O_4_ in all cases, with no secondary or impurity phases detected. As the PVP concentration increases from 0.5 to 2 wt%, a gradual increase in the intensity and sharpness of the diffraction peaks is observed. This trend is particularly noticeable for the (111), (300), and (511) planes, suggesting that higher PVP content contributes to improved structural ordering during the synthesis process. The consistent peak positions across all samples indicate that the lattice structure remains stable and unaltered by the presence of PVP. The enhancement in peak definition reflects a more complete development of the spinel phase as PVP concentration increases, likely due to its influence on directing nucleation and crystal growth [26]. Additionally, the peak profiles indicate improved homogeneity and phase purity at higher PVP levels, as no peak splitting or broad satellite features are observed. The relative intensities of the diffraction peaks remain proportionate, reinforcing that the spinel symmetry is retained across all formulations. The appearance of the (511) plane at higher clarity further supports the formation of well-ordered crystallographic planes. These observations align with the role of PVP as a structure-directing agent that modulates growth kinetics and surface energy during the formation of oxide networks. Overall, the XRD results confirm that the PVP concentration has a pronounced effect on the crystallographic refinement of NiCo_2_O_4_ while preserving its spinel phase integrity [27].

### 3.2. X-Ray Photoelectron Spectroscopy

XPS was carried out to investigate the surface elemental composition and chemical states of the best-performing NiCo-P_1_ sample. The survey spectrum (Figure 2b) confirms the presence of Ni, Co, and O elements, with no impurity peaks detected, indicating high surface purity and successful incorporation of the intended metal oxides. This absence of extraneous signals reinforces the effectiveness of the hydrothermal synthesis route and the role of PVP in preventing contamination during nucleation and growth [28]. High-resolution spectra were acquired for the Ni 2p, Co 2p, and O 1s regions to analyze the oxidation states and chemical environment of the elements. The Ni 2p spectrum (Figure 2c) shows two main spin–orbit doublet peaks of Ni^2+^ and Ni^3+^ and two shakeup satellites. As, shown in Figure 2d, the fitted peaks at 856.05 eV and 873.7 eV are attributed to Ni^3+^, while the peaks at 857.44 eV and 874.8 eV are ascribed to the Ni^2+^, respectively. The peak positions and satellite structures are characteristic of Ni^2+^ and Ni^3+^ species in the spinel NiCo_2_O_4_ framework, confirming the coexistence of multiple valence states that are essential for reversible faradaic redox reactions. The coexistence of these oxidation states enhances the redox-active sites available during charge–discharge processes, contributing to improved specific capacitance and cyclic stability [29]. The high-resolution XPS spectrum of the Co 2p region (Figure 2d) reveals characteristic peaks at 781.24 eV and 782.45 eV, corresponding to Co^3+^ and Co^2+^ species in the 2p_3/2_ region, respectively, along with their spin–orbit counterparts at 797.2 eV (Co^3+^) and 798.7 eV (Co^2+^) in the 2p_1/2_ region. The presence of prominent satellite peaks further confirms the coexistence of mixed-valent cobalt states, which is a characteristic of the spinel NiCo_2_O_4_ structure. This mixed oxidation state (Co^2+^/Co^3+^) facilitates rapid and reversible redox reactions, contributing significantly to the material’s pseudocapacitive behavior [30]. The O 1s spectrum (Figure 2e) is deconvoluted into two primary peaks: the lattice oxygen peak at ~531.5 eV and the surface-adsorbed oxygen or hydroxyl species peak at ~532.7 eV. The strong lattice oxygen peak contribution confirms the integrity of the oxide lattice, indicating that the NiCo_2_O_4_ crystal structure remains stable and well-ordered. Meanwhile, the presence of surface-adsorbed oxygen suggests a high density of oxygen vacancies or surface defects, which can facilitate ion diffusion, increase surface reactivity, and improve pseudocapacitive performance. Collectively, the XPS results confirm that the NiCo-P_1_ sample possesses a rich redox-active surface, mixed-valence metal centers, and oxygen defect features that all synergistically support superior electrochemical behavior in supercapacitor applications [31,32].

### 3.3. Morphological and Elemental Composition

**Figure 3:**
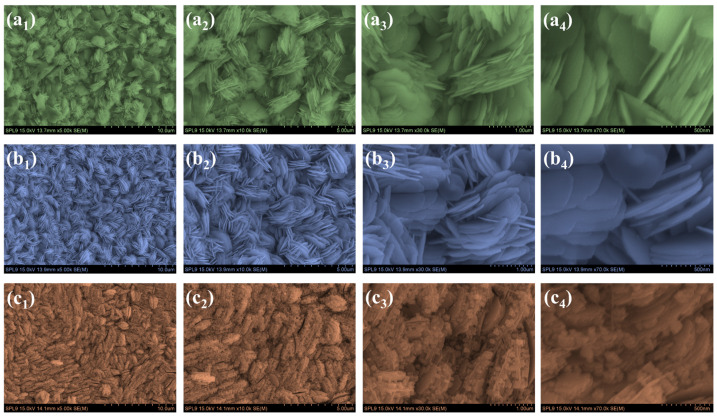
FESEM images of NiCo-P electrodes synthesized with varying PVP concentrations: (**a_1_**–**a_4_**) NiCo-P_0.5_, (**b_1_**–**b_4_**) NiCo-P_1_, and (**c_1_**–**c_4_**) NiCo-P_2_ at increasing magnifications.

The surface morphology of the NiCo_2_O_4_ electrodes, captured through FE-SEM imaging, reveals a striking transformation in nanostructure as the concentration of PVP is systematically varied. Each stage in this transition offers valuable insight into how subtle changes in synthesis parameters can direct nanoscale architecture with profound implications for material performance. In the case of the bare NiCo_2_O_4_ sample (Figure S1(a_1_–a_4_)), synthesized without any PVP, the morphology is dominated by a loosely entangled network of fibrous nanostructures. These wire-like features are irregularly oriented, with minimal cohesion between them. The surface appears discontinuous, marked by significant voids and open spaces. The absence of a templating agent leads to a random, uncontrolled nucleation environment, resulting in a chaotic structure with poor interconnectivity. This morphology, although porous, lacks the structural integrity and organized porosity needed for effective charge transport or mechanical stability [28]. Upon introducing 0.5 wt% PVP (Figure 3(a_1_–a_4_)), the nanostructure undergoes a clear shift from random fibers to more organized, nanosheet-like features. It displays a loosely packed flower-like structure composed of interconnected, petal-like nanosheets. The nanosheets exhibit partial stacking and moderate dispersion, leading to a somewhat open and porous architecture. However, the petals are relatively thicker, and the overall network, while accessible to electrolyte, lacks high density and uniformity. This suboptimal configuration can result in a reduced electroactive surface area, potentially limiting the number of available redox-active sites for charge storage. Consequently, though this structure facilitates ion diffusion, the lower density of nanosheet petals restricts the attainable capacitance and may compromise both rate capability and long-term cycling stability. At 1 wt% PVP, in the sample labeled NiCO-P_1_ (Figure 3(b_1_–b_4_)), the nanostructure reaches a level of exceptional refinement. The surface is uniformly covered with ultra-thin flower-like nanosheets that are precisely stacked and intricately interconnected. These sheets are laterally extended and closely aligned, forming a cohesive, three-dimensional porous network. The architecture exhibits both high surface area and open channels between sheets, offering a well-balanced structure that supports efficient transport phenomena. The spatial distribution of nanosheets is highly uniform, suggesting that PVP at this concentration effectively regulates both nucleation and lateral growth. This controlled environment allows the sheets to develop in an orderly manner without aggregation, resulting in a finely tuned, hierarchical flower-petal framework. However, increasing the PVP content to 2 wt% (NiCO-P_2_) Figure 3(c_1_–c_4_)) introduces excessive surface passivation and hinders optimal crystal growth. The nanosheets become noticeably thicker and begin to stack in a denser, more compact manner. The overall structure turns more granular and less open, with uneven distribution and signs of agglomeration. It appears that excess PVP may saturate the surface and interfere with natural growth pathways, leading to over-capping and a collapse of the open nanosheet network seen in NiCO-P_1_. This results in a loss of structural coherence and reduces the effective surface area. Through this progressive series from random nanowires to structured nanosheets, it becomes evident that the role of PVP is not merely additive but architecturally transformative. The NiCO-P_1_ sample strikes the ideal balance between directed crystal growth and structural openness. The morphology exhibits thin, laterally aligned nanosheets (lower petals) with excellent interconnectivity and hierarchical porosity, attributes that are highly desirable for electrochemical devices. Compared to the disordered structure of the bare NiCo_2_O_4_ and the aggregated form of NiCO-P_2_, the NiCO-P_1_ electrode provides a vastly superior nanostructure capable of supporting rapid ion diffusion and electron transport. It is this precisely engineered nanoscale connectivity that positions NiCO-P_1_ as the most promising candidate for high-performance supercapacitor applications [27,29,32].

**Figure 4:**
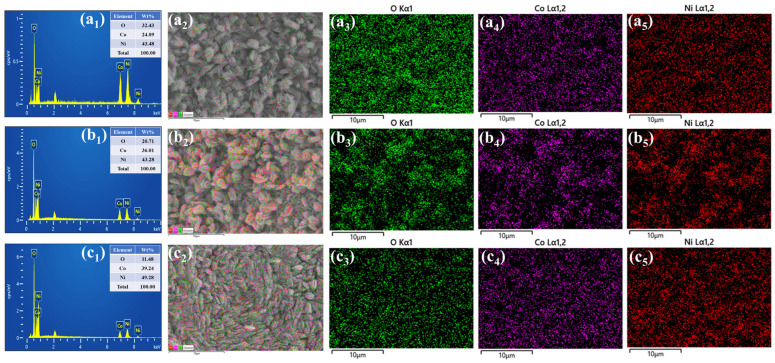
EDS spectra, corresponding SEM overlays, and elemental mapping images of (**a_1_**–**a_5_**) NiCo-P_0.5_, (**b_1_**–**b_5_**) NiCo-P_1_, and (**c_1_**–**c_5_**) NiCo-P_2_ electrodes showing uniform distribution of Ni, Co, and O elements.

Figure 4(a_1_–c_1_) presents the energy-dispersive X-ray spectroscopy (EDS) analysis of the NiCo-P series, along with the reference bare NiCo_2_O_4_ sample (Figure S1(b_1_)), detailing the elemental composition and spatial distribution of Ni, Co, and O. All samples display well-resolved signals corresponding to the constituent elements, confirming the successful formation of the spinel NiCo_2_O_4_ phase. Specifically, the elemental mapping results, along with the corresponding FE-SEM image in Figure S1(b_2_–b_5_) for the bare NiCo_2_O_4_ sample, show a uniform distribution of Ni, Co, and O, indicating good structural homogeneity in the absence of polymer additives. However, the mappings for the PVP-assisted samples, with their respective FE-SEM images in Figure 4(a_2_–a_5_) NiCo-P_0.5_, Figure 4(b_2_–b_5_) NiCo-P_1_, and Figure 4(c_2_–c_5_) NiCo-P_2_, exhibit an even more refined and consistently dispersed elemental distribution, suggesting that PVP played a critical role in enhancing spatial uniformity during synthesis. This improvement in elemental homogeneity implies that PVP effectively modulated nucleation and crystal growth kinetics, preventing phase segregation and promoting the formation of a structurally coherent oxide matrix.

### 3.4. Electrochemical Analysis

**Figure 5:**
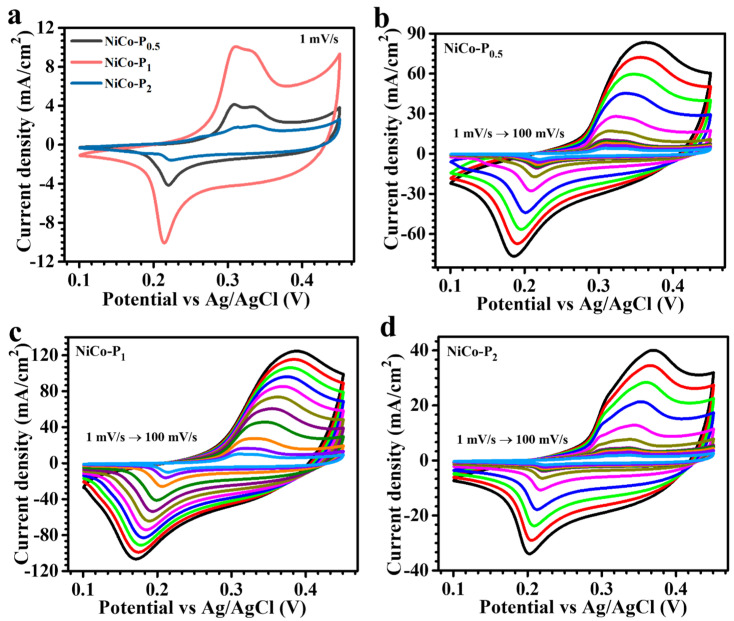
(**a**) Comparative CV curves at 1 mV/s; (**b**–**d**) scan rate-dependent CV curves (1–100 mV/s) of NiCo-P_0.5_, NiCo-P_1_, and NiCo-P_2_ electrodes, respectively.

The electrochemical investigation of the engineered NiCo-P electrodes underscores an exceptional interplay between intricate morphological design and enhanced redox functionality. CV curves (Figure 5a), captured at a controlled scan rate of 1 mV/s within a precisely defined potential range of 0.1 to 0.45 V versus Ag/AgCl, reveal distinctly sharp and symmetrical anodic (~0.21 V) and cathodic (~0.33 V) peaks. These characteristic features are indicative of highly reversible pseudocapacitive processes, arising predominantly from rapid and reversible faradaic transitions involving Ni^2+^/Ni^3+^ and Co^2+^/Co^3+^ redox couples. The efficient and continuous interaction of these active sites with hydroxide ions (OH^-^) within the alkaline electrolyte significantly contributes to robust charge storage performance [33]. Among the studied series, the NiCo-P_1_ electrode, synthesized utilizing 1 wt% PVP, exhibits a pronounced electrochemical superiority. This is distinctly evident from the substantial enclosed area within its cyclic voltammetry profile and heightened redox peak intensities. Such superior electrochemical performance not only indicates enhanced capacitance but also implies rapid charge transfer kinetics and substantially reduced interface impedance. This outcome strongly highlights the critical influence of strategic interface engineering during the PVP-mediated synthesis. The resulting seamless NiCo_2_O_4_ configuration provides an extensive distribution of electroactive sites, facilitating rapid ion mobility and enhancing electrode/electrolyte interactions. Moreover, the nearly symmetric profiles of the redox peaks affirm the quasi-reversible nature of the electrode reactions, suggesting negligible polarization effects and robust electrochemical cycling stability. These redox reactions primarily occur at the electrode surface, characterized by faradaic transformations, and can be briefly represented by the following chemical pathways (1, 2) [34]:

(1) $$Ni{Co}_{2}O_{4}+H_{2}O+{OH}^{-}⇌\;NiOOH+2CoOOH+e^{-}$$

(2) $$CoOOH+{OH}^{-}⇌CoO_{2}+H_{2}O+e^{-}$$

These reactions delineate the synchronized electrochemical behavior of nickel and cobalt oxides, engaging in simultaneous oxidation–reduction cycles facilitated by OH^−^ ion insertion and removal. The optimal concentration of PVP in NiCo-P_1_ evidently maximizes this cooperative redox behavior, simultaneously augmenting electronic conductivity and enhancing structural stability under prolonged cycling conditions.

To elucidate rate capability and electrochemical reversibility further, CV curves were systematically recorded over scan rates from 1 to 100 mV/s (Figure 5b NiCo-P_0.5_, Figure 5c NiCo-P_1_, and Figure 5d NiCo-P_2_). For comparative analysis, pristine NiCo_2_O_4_ (synthesized without PVP) was evaluated under identical conditions, as shown in Figure S1c. All PVP-modified electrodes consistently exhibit pronounced and broad redox peaks, unequivocally indicating pseudocapacitive charge storage primarily governed by redox transitions rather than electrostatic double-layer mechanisms. The distinctly non-rectangular CV shapes strengthen the predominance of faradaic mechanisms, highlighting rapid, reversible surface-confined reactions facilitated by strategic nanostructural engineering. The NiCo-P_1_ electrode distinctly outperformed other variants, demonstrating exemplary electrochemical characteristics due to meticulously balanced parameters of particle distribution, structural homogeneity, and optimal surface architecture. This superior performance is directly linked to the carefully regulated PVP concentration (1 wt%), which ensures a precise equilibrium between particle nucleation and growth. Conversely, lower (0.5%) or higher (2%) PVP contents disrupt this balance by either insufficient particle stabilization or excessive surface encapsulation, leading to suboptimal morphologies characterized by particle aggregation or inhibited growth dynamics [35]. These morphological deviations inherently restrict effective ion and electron transport, adversely affecting electrochemical efficacy. Detailed CV analyses across the range of electrodes elucidate that the NiCo-P_1_ sample uniquely presents intensified redox peak currents accompanied by significantly expanded CV curve areas, a direct consequence of its optimized hierarchical nanosheet morphology. The formation of distinctive, flower-like nanosheet structures with well-defined voids facilitates substantial electroactive surface exposure and enables rapid electrolyte penetration and efficient ion diffusion. This morphology substantially enhances the accessibility of active sites, thereby driving rapid and reversible faradaic processes.

**Figure 6:**
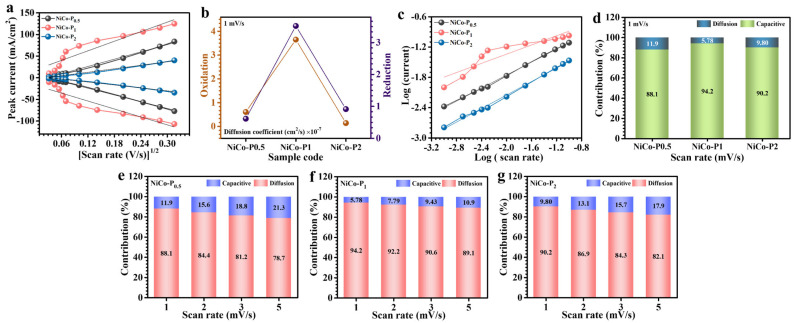
Kinetic analysis of NiCo-P_0.5_, NiCo-P_1_, and NiCo-P_2_ electrodes: (**a**) relationship between peak current and scan rate, (**b**) comparison of oxidation and reduction diffusion coefficient values, (**c**) b-value analysis for redox peaks, (**d**) capacitive vs. diffusion-controlled contribution at 1 mV/s, and (**e**–**g**) charge storage contribution at different scan rates of NiCo-P electrodes.

In-depth evaluation of redox kinetics and ion diffusion mechanisms within NiCo-P electrodes was conducted using CV across varying scan rates. Figure 6a highlights a distinctive linear correlation between the anodic and cathodic peak currents (*i_p_*) and the square root of the scan rate (*v*^1/2^), consistent across all electrode samples. This correlation robustly signifies that the electrochemical reactions within these electrodes predominantly adhere to diffusion-controlled processes, indicative of reversible faradaic kinetics. To quantitatively elucidate the ion diffusion characteristics, the apparent diffusion coefficients (D) were precisely determined via the Randles–Sevcik Equation (3) [36]:

(3) $$D^{1/2}=\frac{i_{p}}{2.69\times 10^{5}\times n^{3/2}\times A\times C\times v^{1/2}}$$

Here, *n* corresponds to the electron number participating in the redox reactions, *A* is the electroactive surface area, *C* is the concentration of redox-active species, and *v* represents the scan rate. Computed diffusion coefficients at a controlled scan rate of 1 mV/s are detailed in Table 1, with comparative visualization provided in Figure 6b. Notably, the NiCo-P_1_ electrode distinctly exhibited the highest diffusion coefficient among the series, underscoring significantly improved ionic mobility and expedited charge transfer kinetics. This enhancement is fundamentally attributed to the meticulously engineered flower-like nanosheet morphology, optimized by the precise incorporation of PVP. Conversely, electrodes with either lower (NiCo-P_0.5_) or higher (NiCo-P_2_) PVP content displayed diminished diffusion coefficients. Specifically, NiCo-P_0.5_ demonstrated inferior performance due to inadequate morphological development and limited electroactive surface exposure, a direct consequence of insufficient surfactant presence. For NiCo-P_2_, excessive PVP promoted surface encapsulation or agglomeration phenomena, thereby obstructing active site accessibility and restraining ion transport [37]. Further, the intrinsic charge storage dynamics of the electrodes were analytically probed using the power-law relationship ($i={av}^{b}$), where the peak current (*i*) is related to the applied scan rate (*v*), with the exponent (b) serving as a fundamental indicator of the operative storage mechanism. Values of b close to 0.5 denote predominantly diffusion-driven faradaic processes, while values approaching 1 imply surface-limited capacitive responses [38]. Linear regression plots of log(*i*) versus log(*v*) (Figure 6c) facilitated the extraction of b-values, systematically compiled in Table 1. The derived b-values ranged between 0.45 and 0.66 for the NiCo-P electrodes, conclusively affirming diffusion-controlled redox processes as the primary charge storage mechanism, although with non-negligible capacitive interactions.

To intricately discern the relative influences of surface-bound capacitive interactions versus diffusion-controlled charge transport, the current response across varied potentials was meticulously deconvoluted through the following mathematical formulation (4) [39]:

(4) $$i\left(V\right)=k_{1}v{+k_{2}v}^{1/2}$$

Herein, the term *k*_1_*v* specifically corresponds to the capacitive contributions predominantly arising at electrode interfaces, while *k*_2_*v*^1/2^ distinctly characterizes the diffusion-dependent component. Constants *k*_1_ and *k*_2_ were accurately determined through rigorous linear regression analysis by plotting *i*(*V*)/*v*^1/2^ against *v*^1/2^, facilitating precise quantification of individual charge-storage mechanisms. Consequently, the total accumulated charge within CV profiles is explicitly divided into (5) [39]:

(5) $$Q_{t}=Q_{s}+Q_{d}$$

where *Q_s_* represents surface capacitive storage and *Q_d_* denotes diffusion-mediated contributions. Implementing this analytical approach at a defined scan rate of 1 mV/s, the capacitive and diffusion-dominated charge proportions for the NiCo-P_0.5_, NiCo-P_1_, and NiCo-P_2_ electrodes were distinctly quantified as 11.9%/88.1%, 5.78%/94.2%, and 9.8%/90.2%, respectively (Figure 6d). These compelling findings underscore the significant enhancement in diffusion-controlled redox activity facilitated by integrating PVP within the NiCo_2_O_4_ framework. Notably, the NiCo-P_1_ electrode exhibited the highest diffusion-driven charge contribution, reaching approximately 94.2% at 1 mV/s, highlighting its superior ionic mobility and highly efficient electrochemical kinetics. This remarkable performance is intrinsically linked to its well-structured hierarchical morphology, characterized by interconnected nanosheets forming an open porous network. Such an architecture substantially enhances electrolyte accessibility and promotes extensive interaction with electroactive surfaces, thus optimizing ionic transport pathways. Additionally, the dependence of charge storage dynamics on scan rates ranging from 1 to 5 mV/s was systematically explored. As demonstrated in Figure 6e NiCo-P_0.5_, Figure 6f NiCo-P_1_, and Figure 6g NiCo-P_2_, a progressive increase in capacitive contributions was observed with rising scan rates for all electrode samples. This trend results from restricted electrolyte-ion diffusion depth at accelerated scanning rates, favoring rapid surface-level charge accumulation. The observed shift towards capacitive-dominated behavior at elevated scan rates emphasizes the critical role of finely tuned electrode morphology in facilitating swift charge transfer and maximized electrochemical efficiency [40,41].

**Figure 7:**
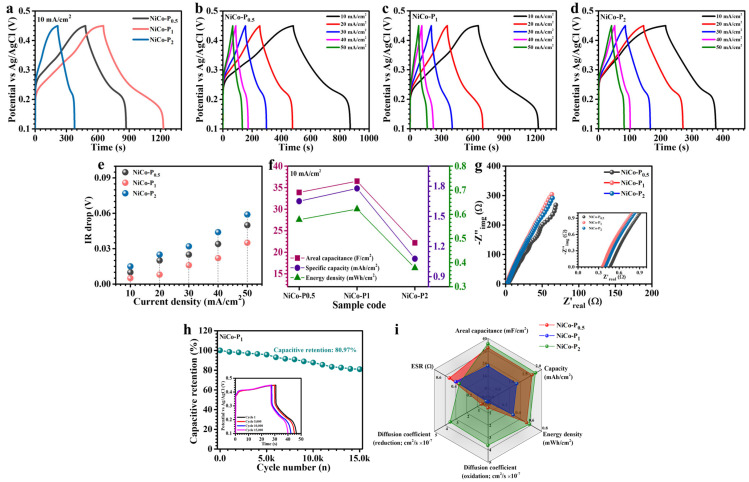
(**a**) Electrochemical performance comparison of NiCo-P electrodes: GCD curve at 10 mA/cm^2^, (**b**–**d**) GCD curves at 10–70 mA/cm^2^ of NiCo-P_0.5_, NiCo-P_1_, and NiCo-P_2_ electrodes, respectively, (**e**) IR drop trend, (**f**) energy storage parameters comparison, (**g**) Nyquist plots and zoomed-in high-frequency region, (**h**) long-term cycling performance of NiCo-P_1_ over 15,000 cycles, and (**i**) radar plot summarizing the multi-parameter electrochemical performance of NiCo-P electrodes.

To meticulously investigate the impact of PVP optimization on the electrochemical performance of NiCo_2_O_4_-based electrodes, GCD and EIS studies were systematically conducted. Comparative GCD curves obtained at a current density of 10 mA/cm^2^ within a defined potential window (0.1–0.45 V) distinctly illustrate electrode-specific performance variations (Figure 7a). Further comprehensive GCD assessments spanning current densities from 10 to 50 mA/cm^2^ were carried out for both pristine NiCo_2_O_4_ (Figure S1d) and PVP-modified electrodes (Figure 7b NiCo-P_0.5_, Figure 7c NiCo-P_1_, and Figure 7d NiCo-P_2_). All electrodes displayed characteristic nonlinear discharge profiles accompanied by evident voltage plateaus, indicative of diffusion-driven faradaic processes typical of battery-type storage mechanisms [42]. Remarkably, the NiCo-P_1_ electrode presented prominently enhanced nonlinear discharge profiles, exhibiting gradual and steady potential declines. This behavior highlights pronounced pseudocapacitive phenomena driven by ion intercalation and reversible surface-confined redox transitions [43,44]. Additionally, the significantly prolonged discharge duration of the NiCo-P_1_ sample compared to NiCo_2_O_4_, NiCo-P_0.5_, and NiCo-P_2_ underscores substantial improvements in energy storage capacity attributed to its intricately optimized nanostructure. Across all electrodes, highly symmetrical charge–discharge patterns underscore excellent coulombic efficiencies and superior electrochemical reversibility, reflective of efficient ionic diffusion kinetics and minimal polarization effects. The NiCo-P_1_ electrode, in particular, demonstrated exceptionally low IR-drop values at the onset of discharge along with a highly symmetric GCD curve, affirming superior electrical conductivity and efficient redox reaction reversibility. Detailed analysis of IR-drop variations across diverse current densities (Figure 7e) further revealed that decreasing current densities consistently lowered IR-drop values, signifying reduced internal resistances, and improved electrode–electrolyte interfacial charge transfer efficiencies. Among all evaluated samples, NiCo-P_1_ consistently exhibited the lowest IR-drop values, corroborating its optimized charge transport dynamics and minimized resistive losses [45]. Further, to accurately quantify electrode performance, key electrochemical metrics, including areal capacitance (C_A_), energy density (ED), and power density (PD), were precisely calculated using integrated formulations tailored for the nonlinear GCD curves (6–8) [46]:

(6) $$C_{A}=\frac{I\times 2\times \int V\left(t\right)dt}{A\times {\left(∆V\right)}^{2}}$$

(7) $$ED=\frac{1}{2\times 3600}\;C_{A}\times {dV}^{2}$$

(8) $$PD=\frac{ED\times 3600}{T_{d}}$$

Here, *I* represents the discharge current, *∫V(t)dt* denotes integration over the discharge curve to accurately capture nonlinear charge storage, *A* indicates electrode surface area, and Δ*V* specifies the operational voltage window. This rigorous calculation approach ensures precise quantification, especially critical for pseudocapacitive systems with distinct nonlinear discharge behaviors governed by faradaic reactions. At 10 mA/cm^2^, areal capacitance values were determined for pristine NiCo_2_O_4_ (24.1 F/cm^2^), NiCo-P_0.5_ (33.9 F/cm^2^), NiCo-P_1_ (36.5 F/cm^2^), and NiCo-P_2_ (22.2 F/cm^2^), with performance metrics comprehensively summarized in Table 2 and comparatively depicted in Figure 7f. Notably, the NiCo-P_1_ electrode demonstrated the highest capacitance among tested samples, a performance directly attributable to its superior hierarchical nanosheet structure, significantly enhancing electrochemically active surface area, electronic conductivity, and accessible active sites. Several synergistic factors underpin this elevated charge storage efficiency: (i) abundant active sites resulting from increased surface area, (ii) optimized electron conduction facilitated by well-controlled NiCo_2_O_4_ deposition, and (iii) enhanced electrolyte penetration via interconnected porous nanosheets. Critically, optimal PVP inclusion effectively mitigates particle aggregation, sustaining structural integrity and preventing nanosheet restacking, thus preserving the vital morphology required for rapid ion transport and efficient charge transfer [34,47]. All electrodes exhibited diminishing capacitance and energy density with increasing current density (Table 2), primarily due to reduced electrolyte-ion penetration depth and diminished utilization of deeper electrode active sites at elevated rates [48]. Despite this, the NiCo-P_1_ electrode sustained remarkable performance retention (approximately 58.1%) at 50 mA/cm^2^, underscoring its robust rate capability and exceptional electrochemical stability.

EIS was performed to elucidate the intrinsic charge transport dynamics and interfacial electrochemical properties of NiCo-P electrodes. Nyquist plots, systematically recorded within a frequency spectrum of 10 kHz to 0.1 Hz in a 2 M KOH electrolyte solution (Figure 7g), provide comprehensive insights into electrolyte solution resistance, electrode interfacial charge transfer mechanisms, and ion diffusion characteristics. Each electrode’s Nyquist profile distinctly exhibits a compressed semicircle in the high-frequency region and an inclined linear portion at lower frequencies, effectively delineating separate electrochemical processes. The intersection point of the high-frequency semicircle on the real impedance axis (Z′) precisely defines the equivalent series resistance (Rs), encompassing contributions from electrolyte ionic resistance, intrinsic material resistance of the electrode, and interfacial contact resistance between the electrode and electrolyte. Simultaneously, the diameter of the semicircle characterizes the charge transfer resistance (Rct), providing a direct indication of the efficacy of faradaic reaction kinetics at the electrode surface [49,50]. Quantitatively, Rs values extracted from the high-frequency intercepts are summarized in Table 1, revealing values of 0.503 Ω, 0.374 Ω, and 0.42 Ω for NiCo-P_0.5_, NiCo-P_1_, and NiCo-P_2_ electrodes, respectively. Significantly, the NiCo-P_1_ electrode exhibits the lowest Rs value (0.374 Ω), clearly indicative of its outstanding electrical conductivity and minimized internal resistance. The superior electrochemical behavior demonstrated by the NiCo-P_1_ electrode is fundamentally linked to its substantially reduced series and charge transfer resistances, which collectively enhance charge transport kinetics, augment rate performance, and significantly elevate overall power delivery efficiency.

To comprehensively assess electrochemical durability, rigorous cycling stability tests were conducted on the NiCo-P_1_ electrode, involving 15,000 continuous charge–discharge cycles executed at an elevated current density of 80 mA/cm^2^ (Figure 7h). Remarkably, the electrode retained approximately 80.97% of its initial capacitance after prolonged cycling, representing a modest capacitance decay of merely ~19.03%. These compelling outcomes underscore the robust long-term electrochemical stability and excellent reversibility inherent in the NiCo-P_1_ electrode’s charge storage processes. The pronounced cycling stability exhibited by the NiCo-P_1_ electrode is fundamentally attributed to its precisely designed hierarchical nanosheet architecture, achieved through optimal PVP concentration during synthesis. This intricate flower-like nanostructure effectively accommodates mechanical strain induced by repeated redox cycling, enhances electrolyte infiltration, and accelerates ionic diffusion within the electrode framework. Consequently, the electrode maintains structural integrity and electroactive accessibility throughout extensive electrochemical utilization. The minor performance deterioration observed after extensive cycling is likely related to ion trapping effects, where electrolyte ions progressively become immobilized in micro-porous regions or accumulate at interfaces, thereby slightly reducing active site accessibility for effective charge exchange. This gradual ion immobilization subtly impedes reversible faradaic reactions, resulting in a moderate reduction of electrode capacitance over prolonged cycling periods [51]. Collectively, these findings reinforce the pivotal role of precise morphological and surface engineering strategies in optimizing the electrochemical properties of pseudocapacitive materials. They vividly highlight how careful structural optimization can profoundly enhance long-term cycling durability, rate capability, and overall charge storage efficiency, critical attributes for practical energy storage applications.

The radar plot provided in Figure 7i serves as an intuitive, multidimensional graphical depiction of critical performance metrics, including areal capacitance, specific capacity, energy density, ion diffusion coefficient, and equivalent series resistance (Rs), for the NiCo-P_0.5_, NiCo-P_1_, and NiCo-P_2_ electrodes. This visualization distinctly reveals the exceptional and consistent electrochemical profile of the optimized NiCo-P_1_ electrode. The extensive and balanced representation across performance parameters underscores the successful consolidation of high capacitance, rapid ionic mobility, and minimized internal resistance into a singular, optimized electrode architecture.

A rigorous comparative assessment highlighting the electrochemical performance of the NiCo-P_1_ electrode in relation to previously established NiCo_2_O_4_-based electrode systems is comprehensively documented in Table S2 [52,53,54,55,56,57,58]. Typically, prior research efforts have reported moderate areal capacitance values, predominantly achieved under limited cycling conditions and lower applied current densities. Contrasting markedly, the present NiCo-P_1_ electrode distinctly surpasses these benchmarks, attaining an exceptional areal capacitance of 36.5 F/cm^2^ at a current density of 10 mA/cm^2^, complemented by remarkable cycling stability extending up to 15,000 cycles. It is important to note that the capacitance values in this study are reported as areal capacitance (F/cm^2^), which is more relevant for practical thin-film applications, rather than gravimetric capacitance (F/g). Our optimized NiCo-P_1_ electrode exhibited an exceptionally high areal capacitance of 36.5 F/cm^2^ at 10 mA/cm^2^, significantly outperforming most literature-reported NiCo_2_O_4_ systems (Table S2). Critically, this elevated performance was realized via an efficient, binder-free hydrothermal synthesis process devoid of complex compositional adjustments or high-temperature procedures. The outstanding results emphasize the efficacy and scalability of the PVP-facilitated synthetic methodology, affirming its suitability for facile production of advanced pseudocapacitive electrodes.

### 3.5. Electrochemical Performance of Asymmetric Supercapacitor Device

**Figure 8:**
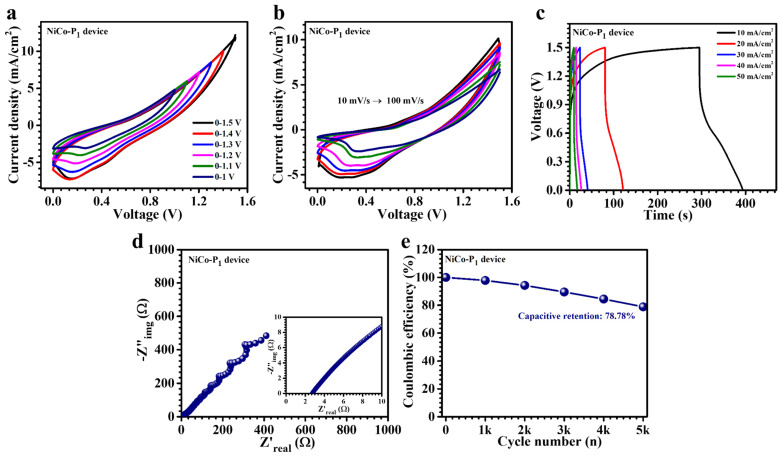
Electrochemical performance of the asymmetric supercapacitor device (NiCo-P_1_//AC): (**a**) CV curves at various voltage windows (0–1.0 to 0–1.5 V) of the asymmetric supercapacitor device (NiCo-P_1_//AC), (**b**) scan rate-dependent CV curves of the device, (**c**) GCD curves at different current densities (10–50 mA/cm^2^), (**d**) Nyquist plot, and (**e**) long-term cycling performance over 5000 cycles.

The practical applicability and performance efficacy of the optimized NiCo-P_1_ electrode were thoroughly examined through the fabrication and characterization of an asymmetric pouch-type supercapacitor device (APSD). Employing a strategic combination of active materials, this device configuration utilized the NiCo-P_1_ electrode as the pseudocapacitive positive electrode to leverage its faradaic charge storage capacity, while activated carbon (AC), known for its superior electric double-layer capacitance, served as the negative electrode. Both electrode materials were appropriately integrated onto nickel foam substrates, ensuring optimal electrical connectivity, mechanical stability, and enhanced performance. To facilitate ionic transport and maintain electrochemical integrity, a separator soaked with a 2 M KOH aqueous electrolyte was incorporated within the device, followed by airtight sealing to prevent environmental contamination and moisture ingress. Comprehensive electrochemical analyses, including CV, GCD, and EIS, were conducted to systematically evaluate the APSD’s performance characteristics. Initial CV studies were performed to ascertain the optimal operating voltage windows for both electrodes individually, revealing stable electrochemical performance ranges of 0.1–0.45 V for the NiCo-P_1_ electrode and −1.0–0.0 V for the AC electrode. The APSD window of 1.5 V was selected based on the working potential ranges of the individual electrodes: NiCo-P_1_ operates within 0.1–0.45 V vs. Ag/AgCl, and the AC electrode, an EDLC-type material, is well established in alkaline electrolytes to function from −1.0 to 0 V vs. Ag/AgCl. Although an independent CV analysis of AC was not conducted in this study, this potential range is widely reported and forms the basis for voltage matching in asymmetric configurations. Thus, the assembled device utilizes a total window of 0 to 1.5 V, which was verified to be electrochemically stable without electrolyte decomposition, as confirmed by stable CV and GCD profiles. Consequently, the overall voltage window of the NiCo-P_1_//AC device was strategically expanded to 1.5 V, effectively maximizing energy storage capacity without risking electrolyte degradation. Further CV investigations across a series of potential windows of 1.0–1.5 V (Figure 8a) demonstrated stable non-rectangular shapes characterized by clear redox peaks, indicative of pseudocapacitive (battery-type) electrochemical behavior. At the scan rate from 10 to 100 mV/s, shown in Figure 8b, CV profiles exhibited enhanced current responses, emphasizing rapid ion diffusion kinetics and superior electrochemical reversibility. The APSD maintained stable electrochemical operation at an extended voltage window of 1.5 V, significantly surpassing typical limitations associated with aqueous electrolyte systems. This extended operational voltage range results from the synergistic interaction between the robust redox properties of the NiCo-P_1_ electrode and the substantial electroactive surface area provided by AC. Note that the CV curves of the APSD display weak redox peaks, which is expected in asymmetric systems combining a pseudocapacitive electrode with an EDLC counterpart. The hybridization of faradaic and capacitive contributions results in distorted or featureless CV curves. Similar behaviors have been reported in the literature and do not indicate poor electrochemical performance. This is supported by the device’s high electrochemical performance, which validates the functional synergy between the NiCo-P_1_ nanosheets and the EDLC-type AC electrode. The device’s pseudocapacitive behavior was further substantiated through detailed GCD assessments (Figure 8c), which presented distinctively nonlinear discharge profiles representative of faradaic reactions. At a moderate current density of 10 mA/cm^2^, the APSD achieved an impressive areal capacitance of 187 mF/cm^2^, complemented by notable energy and power densities of 0.058 mWh/cm^2^ and 1.06 mW/cm^2^, respectively (Table S3). These parameters collectively underscore the device’s ability to efficiently balance high energy storage and rapid power delivery, crucial characteristics for advanced energy storage applications.

EIS measurements provided deeper insights into the internal impedance characteristics of the APSD. The obtained Nyquist plots (Figure 8d) exhibited a compact semicircle in the high-frequency region, indicative of minimal charge transfer resistance at the electrode–electrolyte interface, followed by a linear segment at lower frequencies corresponding to efficient ion diffusion. Notably, the device exhibited an exceptionally low equivalent series resistance (Rs) of 2.72 Ω, underscoring highly efficient charge transport mechanisms facilitated by the optimized electrode nanostructure. This minimal resistance significantly contributes to the enhanced electrochemical kinetics and robust performance of the device. Long-term electrochemical durability, a crucial parameter for practical energy storage applications, was rigorously evaluated through prolonged cycling over 5000 consecutive charge–discharge cycles at an elevated current density of 70 mA/cm^2^, shown in Figure 8e. Remarkably, the APSD demonstrated outstanding capacitance retention of approximately 78.78%. The device’s sustained performance stability can be attributed to its robust, hierarchical nanosheet architecture, effectively mitigating structural degradation due to mechanical stress from repeated electrochemical cycling. This durability is essential for prolonged, high-performance energy storage applications. In brief, the optimized NiCo-P_1_-based APSD demonstrates a highly desirable combination of elevated areal capacitance, expansive operational voltage window, minimal internal resistance, and excellent cycling stability. These superior electrochemical attributes collectively highlight the NiCo-P_1_ electrode as an ideal candidate for advanced flexible and portable supercapacitor systems. Additionally, the outcomes of this study underscore the significance of PVP-assisted synthetic methodologies in achieving precisely tailored electrode architectures designed explicitly for enhanced energy storage performance. The compelling performance exhibited by this APSD emphasizes its potential for integration into next-generation energy storage solutions, marking an essential advancement in the development of high-performance electrochemical devices.

## 4. Conclusions

In this work, we developed a morphology-engineered NiCo_2_O_4_ electrode via a PVP-assisted hydrothermal synthesis strategy, enabling the formation of hierarchical flower-like nanosheets directly on conductive nickel foam substrates. By precisely tuning the PVP concentration, we achieved controlled growth of interconnected nanostructures that maximize electroactive surface area, minimize internal resistance, and facilitate rapid electrolyte ion diffusion. Among all synthesized variants, the NiCo-P_1_ electrode exhibited the most favorable electrochemical characteristics, delivering a high areal capacitance of 36.5 F/cm^2^ at 10 mA/cm^2^, excellent coulombic efficiency, and long-term cycling durability with 80.97% capacitance retention over 15,000 cycles. XPS analysis confirmed the coexistence of Ni^2+^/Ni^3+^ and Co^2+^/Co^3+^ species, contributing to fast and reversible faradaic reactions. Kinetic evaluations, including b-value fitting and capacitive-diffusion deconvolution, demonstrated predominant diffusion-controlled charge storage behavior. EIS and IR-drop analysis revealed low series and charge transfer resistances, further validating the impact of PVP-mediated morphological tuning on charge transport dynamics. Furthermore, when integrated into an asymmetric pouch-type supercapacitor (NiCo-P_1_//AC), the device delivered an extended operating voltage of 1.5 V, high areal capacitance (187 mF/cm^2^), and robust energy and power densities, with 78.78% capacitance retention after 5000 cycles. These findings highlight the critical role of PVP-assisted design in enabling scalable, binder-free, and thermally benign synthesis of advanced transition metal oxide electrodes. This strategy offers a promising route toward the realization of next-generation flexible, high-performance, and cost-effective electrochemical energy storage systems.

## Figures and Tables

**Table 1:** Calculated diffusion coefficients (D), b-values, and series resistances (Rs) for NiCo-P_0.5_, NiCo-P_1_, and NiCo-P_2_ electrodes.

Sample Code	Diffusion Coefficient (cm^2^/s) × 10^−7^		b-Value	R_s_ (Ω)
	Oxidation	Reduction		
NiCo-P_0.5_	0.608	0.615	0.64	0.503
NiCo-P_1_	3.65	3.52	0.45	0.374
NiCo-P_2_	0.14	0.15	0.66	0.42

**Table 2:** Comparison of electrochemical performance parameters: areal capacitance (CA), areal capacity (C), energy density (ED), and power density (PD) at varying current densities.

Sample Code	I (mA//cm^2^)	C_A_ (F/cm^2^)	C (mAh/cm^2^)	ED (mWh/cm^2^)	PD (mW/cm^2^)
NiCo-P_0.5_	10	33.9	1.651	0.578	4.59
	20	31.5	1.532	0.536	9.24
	30	29	1.413	0.494	13.45
	40	20.9	1.016	0.356	16.74
	50	17.1	0.833	0.292	20.00
NiCo-P_1_	10	36.5	1.778	0.622	3.36
	20	31.8	1.548	0.542	6.59
	30	29.3	1.429	0.500	9.05
	40	23.5	1.143	0.400	11.64
	50	21.2	1.032	0.361	12.96
NiCo-P_2_	10	22.2	1.079	0.378	4.67
	20	20.6	1.000	0.350	10.79
	30	17.1	0.833	0.292	15.75
	40	16.3	0.764	0.278	19.61
	50	13.9	0.675	0.236	26.25

## Data Availability

The original contributions presented in the study are included in the article/Supplementary Materials, further inquiries can be directed to the corresponding author.

## Supplementary Materials

The following supporting information can be downloaded at: https://www.mdpi.com/article/10.3390/polym17131802/s1, Figure S1: (a_1_–a_4_) FESEM images of the pristine NiCo_2_O_4_ electrode at increasing magnifications, (b_1_–b_5_) EDS spectrum and corresponding elemental mapping images confirming the uniform distribution of Ni, Co, and O, (c) CV curves at various scan rates; and (d) GCD curves at different current densities. Table S1: Energy storage parameters of pristine NiCo_2_O_4_ electrode at various current densities, indicating moderate charge storage performance compared to PVP-assisted counterparts. Table S2: Comparison of electrochemical performance of NiCo_2_O_4_-based electrodes synthesized via various methods, energy storage performance, with the present PVP-assisted NiCo_2_O_4_ electrode. Table S3: Electrochemical performance of the NiCo-P_1_//AC asymmetric pouch-type supercapacitor device at various current densities.
