# Supplementary material for: Polyvinylpyrrolidone-Functionalized NiCo2O4 Electrodes for Advanced Asymmetric Supercapacitor Application
Source: Polymers (Basel). 2025 Jun 28;17(13):1802. doi: 10.3390/polym17131802 (PMC12252293; doi:10.3390/polym17131802)
Supplement: Supplementary file 1 [file polymers-17-01802-s001.zip › polymers-3724227-supplementary.pdf]

Supplementary File

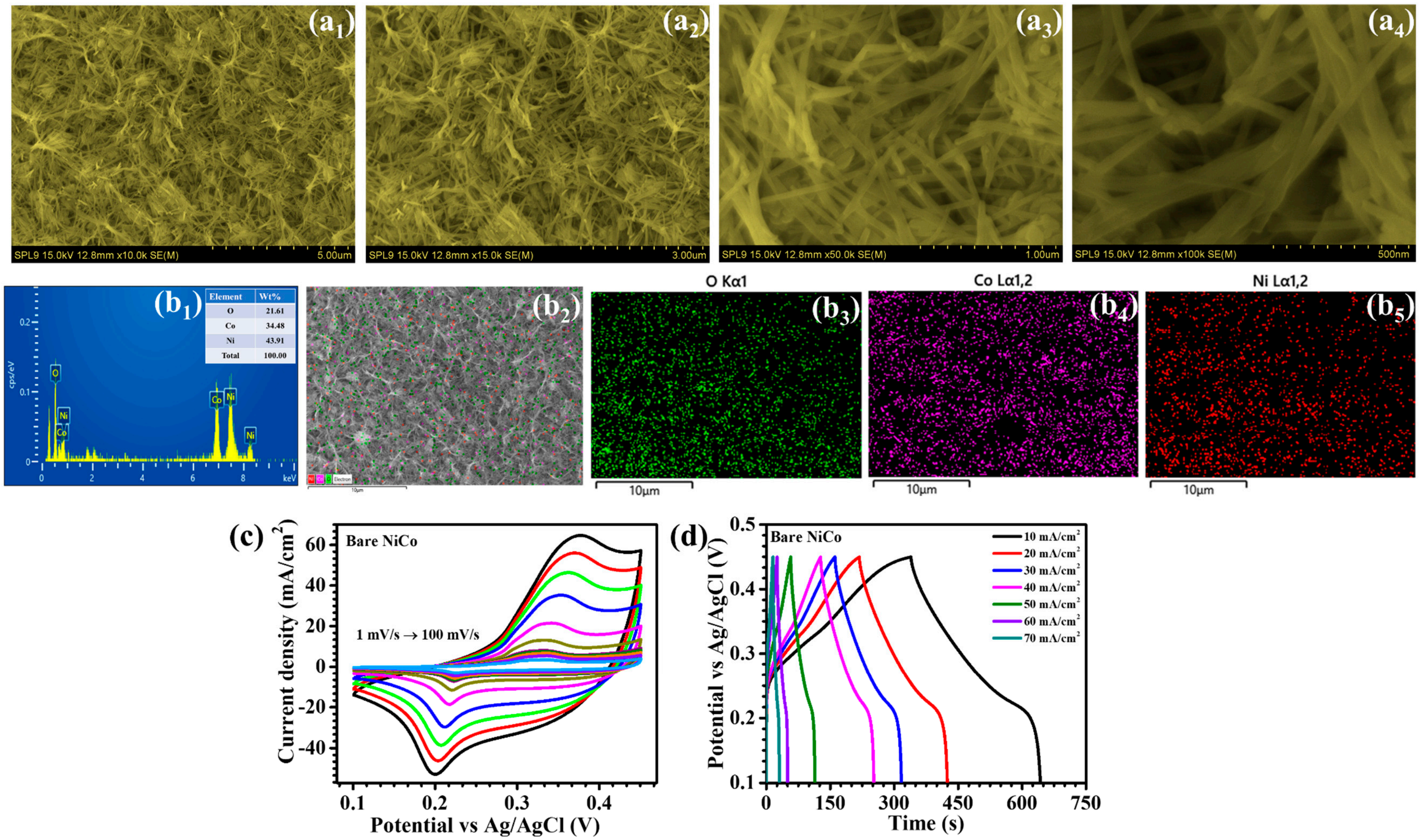

Figure S1. (a_1–a_4) FESEM images of the pristine $NiCo_2O_4$ electrode at increasing magnifications, (b_1–b_5) EDS spectrum and corresponding elemental mapping images confirming the uniform distribution of Ni, Co, and O, (c) CV curves at various scan rates; and (d) GCD curves at different current densities.

Table S1. Energy storage parameters of pristine $NiCo_2O_4$ electrode at various current densities, indicating moderate charge storage performance compared to PVP-assisted counterparts.

**Sample code**	**I (mA/cm²)**	**C_A (F/cm²)**
**Pristine $NiCo_2O_4$**	10	24.1
	20	21.2
	30	14.28
	40	13.37
	50	11.88

Table S2. Comparison of electrochemical performance of $NiCo_2O_4$-based electrodes synthesized via various methods, energy storage performance, with the present PVP-assisted $NiCo_2O_4$ electrode.

Sr. No.	Material	Synthesis method	Current	Electrolyte	Areal capacitance	Cycle stability	Ref.
1.	$NiCo_2O_4$@RVC	Hydrothermal	-	0.5 M KOH	2.45 F/cm²	10,000 cycles (96.7% stability)	[52]
2.	NiCo2O4	Hydrothermal	1 A/g	2 M KOH	1584 F/g	1000 cycles	[53]
3.	NiCo2O4	Microwave assisted	1 mA/cm²	2 M KOH	358.3 mF/cm²	6,000 cycles (78% stability)	[54]
4.	NiCo 2O4 @NiCo 2O 4 core/shell	Solution-based	2 mA/cm²	2 M KOH	1.55 F/cm²	4,000 cycles (98.6% stability)	[55]
5.	NiCo2O4	pulsed laser ablation	1 A/g	3 M KOH	1650 F/g	12,000 cycles (91.78% stability)	[56]
6.	NiCo2O4	Solution-based	1.8 mA/cm²	2 M KOH	3.51 F/cm²	3,000 cycles (93.3% stability)	[57]
7.	Co_3O_4@$NiCo_2O_4$	Hydrothermal	-	2 M KOH	2.04 F/cm²	75,000 cycles (83.7% stability)	[58]
**8.**	**$NiCo_2O_4$-PVP**	**Hydrothermal**	**10 mA/cm²**	**2M KOH**	**36.5 F/cm²**	**15, 000 cycles (80.97% stability)**	**This work**

Table S3: Electrochemical performance of the NiCo-P_1//AC asymmetric pouch-type supercapacitor device at various current densities.

Sample code	I (mA)	C_A (mF/cm^2)	C (mAh/cm^2)	ED (mWh/cm^2)	PD (mW/cm^2)
NiCo-P_1 device	10	187	0.039	0.058	1.06
	20	36	0.007	0.011	0.50
	30	8	0.002	0.003	0.25
	40	5	0.001	0.002	0.23
	50	4	0.001	0.001	0.24
